# Tau Oligomers: The Toxic Player at Synapses in Alzheimer’s Disease

**DOI:** 10.3389/fncel.2015.00464

**Published:** 2015-12-02

**Authors:** Marcos J. Guerrero-Muñoz, Julia Gerson, Diana L. Castillo-Carranza

**Affiliations:** ^1^Mitchell Center for Neurodegenerative Diseases, University of Texas Medical Branch, GalvestonTX, USA; ^2^Departments of Neurology, Neuroscience and Cell Biology, University of Texas Medical Branch, GalvestonTX, USA

**Keywords:** Alzheimer’s disease, tau oligomers, Aβ oligomers, synapsis, dendrites

## Abstract

Alzheimer’s disease (AD) is a progressive disorder in which the most noticeable symptoms are cognitive impairment and memory loss. However, the precise mechanism by which those symptoms develop remains unknown. Of note, neuronal loss occurs at sites where synaptic dysfunction is observed earlier, suggesting that altered synaptic connections precede neuronal loss. The abnormal accumulation of amyloid-β (Aβ) and tau protein is the main histopathological feature of the disease. Several lines of evidence suggest that the small oligomeric forms of Aβ and tau may act synergistically to promote synaptic dysfunction in AD. Remarkably, tau pathology correlates better with the progression of the disease than Aβ. Recently, a growing number of studies have begun to suggest that missorting of tau protein from the axon to the dendrites is required to mediate the detrimental effects of Aβ. In this review we discuss the novel findings regarding the potential mechanisms by which tau oligomers contribute to synaptic dysfunction in AD.

## Introduction

Alzheimer’s disease (AD) is a devastating progressive neurodegenerative condition and the most common cause of dementia among the elderly. The disease is characterized by memory loss and cognitive impairment, and eventually the inability to perform daily life activities. Currently, available treatments for AD only provide relief of symptoms with no effect on the course of the disease. As the longevity of the worldwide population increases, the amount of people susceptible to AD will continue to rise (Reitz and Mayeux, 2014). After decades of research, the precise underlying cause or causes of sporadic AD remain unknown. Therefore, there is an urgent need to understand the pathological mechanisms involved in AD to develop effective treatments.

The profound neuropathological changes to synaptic communication seem to be responsible for cognitive decline and memory dysfunction, the most striking symptoms of AD. However, a great deal of research is needed to come to a complete understanding of the mechanism by which these symptoms develop. Analysis of AD brain cases have revealed synaptic degeneration, neuronal loss and accumulation of extracellular amyloid plaques and intracellular neurofibrillary tangles (NFTs) composed mainly of fibrillar amyloid β peptide (Aβ) and tau protein, respectively (Serrano-Pozo et al., 2011a). For over two decades amyloid plaques were considered to be the primary cause of AD (Hardy and Allsop, 1991). However, amyloid plaque deposition does not correlate with cognitive impairment observed in AD patients.

While amyloid pathology lies upstream of tau pathology (Oddo et al., 2003; Small and Duff, 2008), growing evidence indicates that tau pathology drives cognitive decline in AD (Murray et al., 2015), providing an explanation for the lack of connection between the staging of amyloid plaques and disease symptoms. Tau pathology has thus been considered a secondary amyloidosis in the progression of AD, but the relationship between Aβ and tau is still contentious. The main function of tau is to stabilize microtubules within the neurons. In AD, tau undergoes post-translational modifications that affect the affinity of tau to the microtubule, leading to tau self-association and the eventual formation of NFTs. It has been established that soluble forms of Aβ, but not fibrils (Kayed et al., 2003; Baglioni et al., 2006; Haass and Selkoe, 2007; Shankar et al., 2007; Walsh and Selkoe, 2007), correlate with the onset of the disease only in the presence of tau, suggesting that the latter mediates Aβ toxicity (Lue et al., 1999; McLean et al., 1999; Wang et al., 1999). However, though NFTs correlate better with cognitive decline and neuronal loss (Braak and Braak, 1991b; Arriagada et al., 1992; Gomez-Isla et al., 1997; Giannakopoulos et al., 2003) than amyloid deposits, they do not seem to be the most toxic tau aggregates in disease, with many studies supporting the hypothesis that soluble oligomers drive tau toxicity (Maeda et al., 2006, 2007; Patterson et al., 2011; Lasagna-Reeves et al., 2012b). The deleterious effects of tau pathology may be partly due to a gain of toxic function. The discovery that mutations in the gene encoding tau (MAPT) lead to neurodegeneration strongly supports this hypothesis. Although no mutations in the MAPT gene have been found in AD, they are associated with familial frontotemporal dementia (FTDP-17) reviewed by Goedert et al. (1999), resulting in tau’s inability to bind microtubules and subsequent aggregation into oligomers and NFT’s. Since microtubules are important components of axonal processes, the loss of tau function affects neuronal stability and impairs axonal transport. However ablation of tau in mice does not induce neurological deficits or cell death but instead makes the tau knockout more resistant to seizures (Roberson et al., 2007), suggesting that the pathogenesis of tau is not due solely to a loss of function.

Whether or not the loss of tau function leads to neuronal dysfunction is still in debate. However, there is a large body of evidence demonstrating that aggregated tau acquires a toxic function in which tau oligomers are clearly implicated as driving the mechanism.

In this review, we summarize novel findings regarding the role of tau oligomers at the synapse and their interaction with other amyloid proteins in mediating cognitive decline in AD.

## Synaptic Effects Of Insoluble Aggregates In Ad

Synaptic plasticity is thought to be the route by which learning and the acquisition of new memories occurs. In AD, marked synapse loss underlies cognitive deficits that appear to depend upon neurodegenerative processes induced by Aβ and tau. Postmortem human brain samples have been found to show gliosis and oxidative stress in the vicinity of amyloid plaques and NFT’s that may contribute to synaptic changes (McLellan et al., 2003; Ingelsson et al., 2004; Serrano-Pozo et al., 2011b). The overexpression of Aβ in mice revealed neurite degeneration after plaque formation (Meyer-Luehmann et al., 2008). However, therapeutic approaches in AD mouse models suggested that plaques are inert and an increase in this metastable aggregate is not associated with neurological deficits (Cheng et al., 2007), but rather is beneficial since cognitive function was improved in mice (Jankowsky et al., 2003, 2005; Lesne et al., 2008). Using the Tg2576 mouse model, we found that removal of tau oligomers by immunotherapy shifted the Aβ aggregation pathway to amyloid plaques, while improving cognition in mice (Castillo-Carranza et al., 2015). These findings could explain the presence of amyloid plaques in individuals without clinical symptoms of AD, thus termed high pathology controls or non-demented with AD neuropathology (NDAN) subjects (Bjorklund et al., 2012) and unsuccessful clinical trials even after removing amyloid plaques (Cappai and Barnham, 2008; Hardy, 2009).

Neurites surrounding plaques often contain phosphorylated tau aggregates (Woodhouse et al., 2005; Serrano-Pozo et al., 2011a). During the course of AD, tau is hyperphosphorylated and accumulates into fibrillar aggregates in the somatodendritic compartment (Spillantini and Goedert, 2013). NFTs have historically been considered the main hallmark in tauopathies, including AD (Braak and Braak, 1991a,b, 1996). However, NFT-containing neurons have been shown to be functionally intact *in vivo* (Kuchibhotla et al., 2014). A comparative analysis of AD cases versus high-pathology control or NDAN subjects revealed no significant differences in levels of NFTs, rather showing that increased levels of phosphorylated tau in the synaptic compartment were associated with dementia (Perez-Nievas et al., 2013). While signaling cascades involved in long-term potentiation and memory are not affected by NFTs (Kuchibhotla et al., 2014) postmortem analysis of brains from people with mild cognitive impairment showed that cognitive symptoms correlate with pre-fibrillar tau rather than NFT’s (Vana et al., 2011; Mufson et al., 2014). Further supporting *ex vivo* evidence for the importance of a tau aggregation intermediate in neurodegeneration, tau transgenic animal models acquire behavioral deficits, synaptic dysfunction, and cell death in the absence of NFT formation (Wittmann et al., 2001; Andorfer et al., 2003; SantaCruz et al., 2005; Spires et al., 2006; Berger et al., 2007; Yoshiyama et al., 2007; Cowan et al., 2010). Furthermore, upon suppression of tau in tauopathy transgenic models, mice show cognitive improvement in spite of continued presence of NFTs (SantaCruz et al., 2005; Sydow et al., 2011). Moreover, electrophysiological impairment and structural degeneration to neurons do not depend on the presence of NFTs (Rocher et al., 2010; Crimins et al., 2012). The observation that cell death occurs in disease prior to the formation of NFTs, suggests that pre-filamentous forms of tau confer toxicity before fibrillization (Gomez-Isla et al., 1997; Terry, 2000; van de Nes et al., 2008).

## TAU Oligomers As The Toxic Protein Species In Disease

Protein misfolding is the initial step in the aggregation pathway of both Aβ and tau. Post-translational modifications and the formation of disulfide bridges increase the ability of both proteins to self-aggregate into oligomers (Chirita et al., 2005; Sahara et al., 2007). Evidence suggests that tau monomer is first converted to an oligomeric state prior to the formation of fibrils (Ruschak and Miranker, 2009; Lasagna-Reeves et al., 2010; Lee et al., 2011). *In vitro*, tau aggregation does not occur spontaneously but the addition of polyanionic compounds and free fatty acids induce fibril formation (King et al., 2000; Barghorn and Mandelkow, 2002; Chirita et al., 2003; von Bergen et al., 2005). These various structures differ not only in aggregation state, but also in their toxic effects.

Growing data suggest that prefilamentous forms of tau, specifically oligomers, are neurotoxic (Patterson et al., 2011; Lasagna-Reeves et al., 2012a). Tau oligomers have been isolated at very early stages of the disease, prior to the onset of the clinical symptoms (Maeda et al., 2006; Lasagna-Reeves et al., 2012b). By atomic force microscopy (AFM), tau oligomers display a spherical morphology that corresponds with two or more molecules of tau, ranging between 6 and 20 nm (Sahara et al., 2008). These are dynamic structures that become β-sheet rich (Lasagna-Reeves et al., 2010). In brain samples from AD cases, tau oligomers were found at a fourfold higher concentration compared to healthy control samples (Himmelstein et al., 2012). In AD, tau is abnormally phosphorylated at multiple positions. However this may not be a requirement for tau to be able to form oligomers and become toxic.

In addition to AD, tau oligomers were identified in progressive supranuclear palsy (PSP), dementia with Lewy bodies (DLB) as well as Huntington’s diseases cases (Gerson et al., 2014; Sengupta et al., 2015; Vuono et al., 2015). Thus, the presence of tau oligomers in several tauopathies prompted the hypothesis that tau oligomers follow a common mechanism of toxicity between diseases. However, little is known about the properties of tau oligomers and the mechanism by which they lead to cell loss.

Many studies have demonstrated the toxicity of tau oligomers when applied extracellularly to cultured neuronal cells, leading to tau uptake into the cell and increased intracellular calcium levels. In mice, the injection of tau oligomers induces mitochondrial abnormalities and synaptic dysfunction (Berger et al., 2007; Lasagna-Reeves et al., 2011).

## Cross-Talk Of TAU Oligomers And Other Amyloids At The Synapse

**FIGURE 1:**
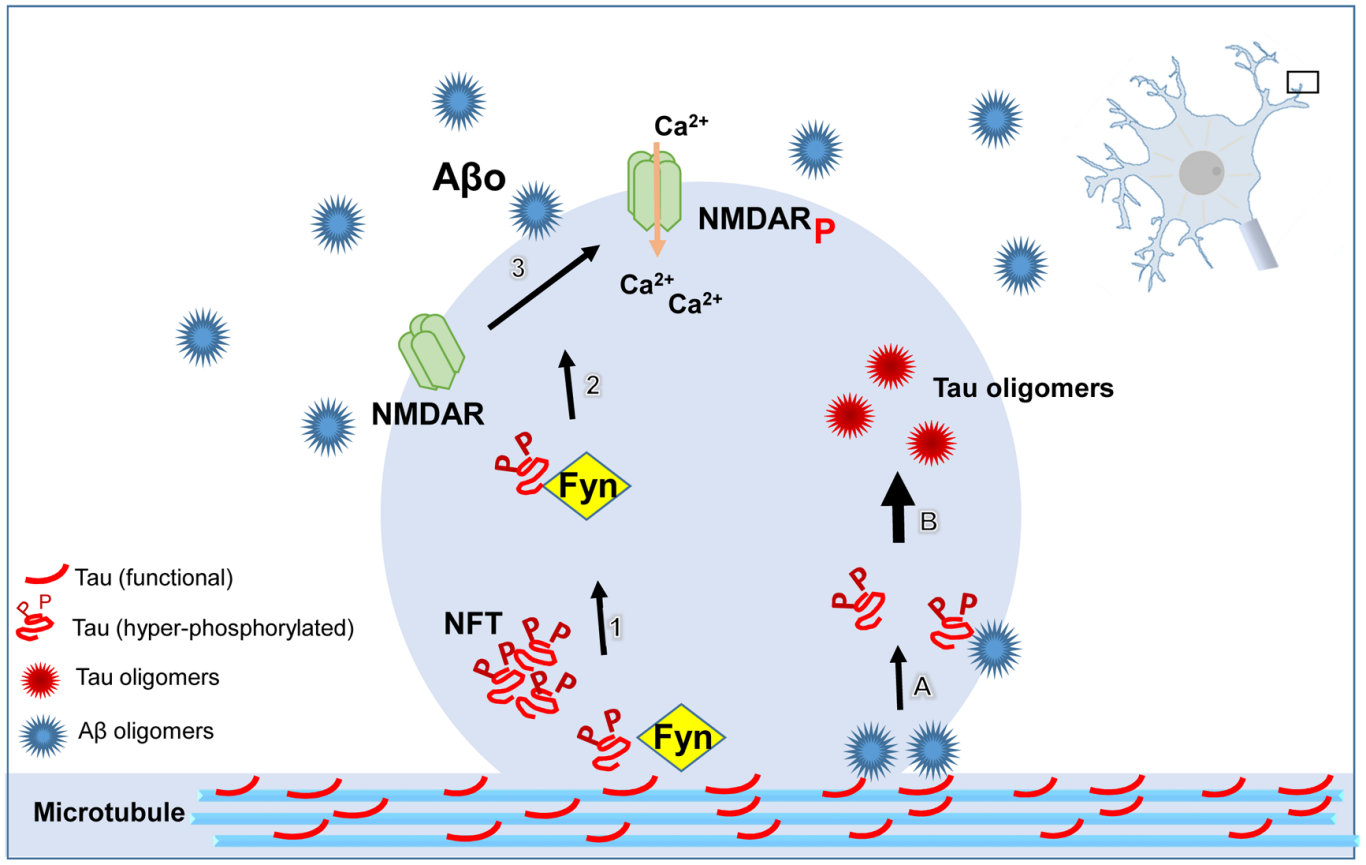
**Schematic illustrating the pathological role of tau at dendritic spines. ß-amyloid (Aβ) oligomers directly or indirectly lead to the dystrophic changes in neurites mediated by tau.** Hyperphosphorylated tau targets the kinase, Fyn, to the postsynaptic compartment. Fyn phosphorylates NR2B, a subunit of the N-methyl-D-aspartate receptor (NMDAR), resulting in the over-activation of NMDAR, followed by increased concentration of Ca^2+^ in the cytoplasmic compartment. Aβ oligomers (Aβo) seed tau misfolding and aggregation by direct interaction resulting in tau oligomer formation at dendritic spines. All of these pathways converge in the aggregation of tau protein, spine loss, and consequently, cognitive impairment.

Growing evidence suggests that the accumulation of tau oligomers at the synapse may be critical for neurodegeneration. We have shown that recombinant tau oligomers display amnesic effects and synaptic dysfunction when administered intracranially to wild-type mice (Lasagna-Reeves et al., 2011, 2012b). It appears as though a redistribution of pathological tau from the axon to the cell body and dendrites is responsible for spine loss observed in disease (Zempel et al., 2010). In AD patients, Aβ binds preferentially to neuronal dendrites promoting tau missorting (Zempel and Mandelkow, 2012). It is well-established that aggregated Aβ contributes to tau phosphorylation and mislocalization (Gotz et al., 2001; Ferrari et al., 2003; De Felice et al., 2008; Ittner et al., 2010; Chabrier et al., 2012). However, a growing body of evidence suggests that Aβ induces tau pathology by direct interaction in a prion-like manner. In the prion field, the acquisition of β-sheet conformation by a prion protein allows it to seed the misfolding and aggregation of other prion molecules, reviewed by Jucker and Walker (2011). The pathologic similarities between prion disease and AD suggest that it might also be inducible in a prion-like manner. *In vitro* assays have shown that Aβ oligomers can seed tau oligomerization, providing evidence that this phenomenon may occur *in vivo* (Lasagna-Reeves et al., 2010). The induction of tau misfolding in transgenic mice overexpressing amyloid precursor protein (APP) (Castillo-Carranza et al., 2015) and mice infused with brain extract from aged APP23 transgenic mice (Bolmont et al., 2007), suggest that Aβ can seed tau oligomerization *in vivo* as well (**Figure 1**). Importantly, it is possible that a direct interaction between the two proteins may be involved in the induction of synaptic dysfunction as tau and Aβ coexist within synaptic compartments in AD brain (Hoover et al., 2010; Ittner et al., 2010; Zempel et al., 2010; Tai et al., 2012; Miller et al., 2014). However, the complexity and diversity of amyloid aggregates has made the elucidation of the interaction between the two proteins difficult. In humans, a 56-kDa Aβ aggregate, called Aβ^∗^56 seems to have a pathogenic role specifically during the asymptomatic phase of AD (Handoko et al., 2013). Notably, Aβ^∗^56 correlates with the pathological form of tau (Lesne, 2013) whereas Aβ dimers isolated from AD samples trigger endogenous tau hyperphosphorylation followed by neuritic degeneration of cells in culture (Jin et al., 2011). Different Aβ assemblies including Aβ^∗^56 are prominent in the synaptic terminals of AD patients (Sokolow et al., 2012). Recently, we provided evidence for the interaction of tau oligomers and Aβ peptide in the Tg2576 mouse. The reduction of tau oligomers by immunotherapy ameliorated memory deficits, implying a role for tau in mediating cognitive decline in aged mice overexpressing APP. Moreover, a marked reduction of Aβ^∗^56 and increase of trimers suggest that the removal of tau oligomers modulates Aβ levels (Castillo-Carranza et al., 2015). It seems likely that the increase in trimers in treated mice may be a consequence of Aβ^∗^56 disassembly which has been suggested to be comprised of four Aβ trimers (Lesne et al., 2006). However, reduction of Aβ alone by immunotherapeutic approaches was not sufficient to improve cognition in mice displaying tau pathology, whereas reduction of both pathologies did confer benefits (Oddo et al., 2006) providing support for a synergistic relationship between Aβ and tau in which tau induces toxicity downstream of Aβ.

While Aβ and tau aggregates are the two main pathological hallmarks of AD, Lewy bodies comprised of α-synuclein protein are found in more than half of sporadic AD cases studied (Hamilton, 2000). Importantly, a recent study showed that toxic, non-fibrillar α-synuclein is significantly elevated in AD cases absent Lewy body pathology (Larson et al., 2012). In its native state, α-synuclein is found at the synapse where it promotes neurotransmitter release (Burré et al., 2010), highlighting its potential importance in synaptotoxicity in AD. Moreover, elevated soluble α-synuclein was associated with a decrease in presynaptic vesicle proteins in AD brains (Larson et al., 2012). These results combined with evidence that oligomeric tau and α-synuclein interact and co-aggregate in disease (Sengupta et al., 2015) suggests that the two proteins may act in a toxic synergistic mechanism at the synapse in AD.

## Role Of TAU In Synaptogenesis

Tau protein promotes neurite outgrowth and is differentially expressed and phosphorylated in the developing brain. During periods of neurite growth, high levels of tau phosphorylated at Ser202 and Thr205 (recognized by AT8 antibody) are seen similarly to during Alzheimer’s conditions, while levels are dramatically reduced when neurites are stabilized and synaptogenesis occurs, corresponding to tau levels and phosphorylation state in healthy adult brain (Brion et al., 1994; Rösner et al., 1995; Riederer, 2001). Collectively, these results suggest that study into the normal function of tau protein may be critical to understanding the synaptic dysfunction due to tau abnormalities in AD. The decrease in synaptic function may be due partially to an overall decrease in synaptogenesis in AD. Cell adhesion molecules such as Nectin-3 are important for both synaptic plasticity and synaptogenesis. Expression of human tau protein as well as tau injections in mice were shown to be associated with a decrease in levels of Nectin-3 in brain regions of importance to memory and cognition (Maurin et al., 2013). Moreover, neurons expressing neuronal nitric oxide synthase that acts as a messenger for synaptogenesis are particularly prone to neurodegeneration in AD brains and the protein was found to colocalize with NFTs (Thorns et al., 1998). A recent study showed that levels of neurogenesis are significantly lowered in the Htau mouse model overexpressing human wild-type tau in a mouse tau knockout background, providing evidence that tau aggregation alone may decrease neurogenesis and synaptogenesis (Komuro et al., 2015).

## TAU Oligomers And Synaptic Dysfunction

The characterization of tau aggregates and potential routes of tau spreading has led to important results indicating that tau oligomers can be found in a large percentage of pre-synaptic and post-synaptic compartments in AD, suggesting a toxic role for tau oligomers in synaptic transmission (Tai et al., 2014). Synaptic communication occurs at dendritic spines. Thereby, reductions in spine number or morphological changes would be expected to contribute to synaptic dysfunction and cognitive deficits. Studies have shown that Aβ oligomers interact with tau, inducing its translocation to synaptic spines (Frandemiche et al., 2014). Dendritic spines present with various morphologies can drastically affect their functionality. Spines found to have particularly large post-synaptic densities are believed to provide for stable synaptic connections for memory formation. We have found that a reduction of tau oligomers in the Tg2576 AD mouse model is associated with a significant increase in mushroom-shaped spines with large postsynaptic densities (Castillo-Carranza et al., 2015). Moreover, in Htau mice the levels of tau correlate with cognitive deficits, decrease in long term potentiation, lowering of synaptic proteins, and a reduction in the level of mushroom-shaped spines as well as an increase in amount of thin spines (Polydoro et al., 2009; Dickstein et al., 2010; Alldred et al., 2012). A mouse model overexpressing both Aβ and wildtype tau exhibited a synergistic toxic effect to dendritic spines that was greater than effects of the expression of either protein alone (Chabrier et al., 2014). While these results highlight the toxic effect of tau pathology at the synapse, some studies have shown that Aβ can induce synaptic dysfunction, spine loss, and changes to spine morphology independently of tau (Shahani et al., 2006; Tackenberg and Brandt, 2009; Tackenberg et al., 2013). On the other hand, tau aggregation alone has been shown to be sufficient to cause synaptic detriment. The injection of human tau blocked synaptic transmission of squid axons (Moreno et al., 2011). A comparative analysis of synapses showed that only AD brains but not controls, contained tau phosphorylated at serines 396/404 in a greater number of postsynaptic than presynaptic sites (Tai et al., 2012). In contrast, this type of tau is accumulated in the presynapse of the entorhinal cortex from aged P301L mice, a transgenic overexpressing human mutant tau (Harris et al., 2012). The accumulation of phosphorylated P301L mutated tau is accompanied by disruption of synaptic transmission and impaired glutamate receptor subunit GluA1, GluA2/3, and NR1 trafficking to the postsynaptic density (Hoover et al., 2010). Overexpression of P301L tau in rTg4510 mice causes synaptic dysfunction and loss of synapses (Rocher et al., 2010; Crimins et al., 2011, 2012, 2013; Kopeikina et al., 2011, 2013). A recent study by Decker et al., showed that pre-NFTs—likely comprised largely of tau oligomers—cause pre- and postsynaptic morphological changes (a gain of toxic function) at the mossy fibers located in the CA3 brain region in transgenic mice expressing the aggregation prone ΔK280 (Tau^RDΔ^) mutant human Tau (Decker et al., 2015). Interestingly, examination of tau knockout mice revealed normal synaptic plasticity, but weak synaptic transmission comparable to mice Tau^RDΔ^. Previous studies have shown that missorting of tau to the somatodendritic compartment leads to retraction of mossy fibers from CA3 in hibernating ground squirrels, in a process which seems to be reversible suggesting a physiological role of Tau in mossy fiber plasticity (Arendt et al., 2003).

Taken together, these studies suggest an important role for tau within both the pre- and postsynapse, suggesting that when tau misfolds and aggregates into oligomers in disease it may cause synaptic dysfunction.

## TAU Oligomers Induce Abnormal Synaptic Plasticity

Recently, the function of tau has been expanded to include a role in synaptic plasticity. Studies showed that knocking out tau *in vivo* eliminates long term depression (LTD) in the CA1 of the hippocampus. LTD describes an activity-dependent reduction or weakening of synaptic contacts. Tau phosphorylation induced by Aβ-mediated NMDA receptor activation is associated with an increase in LTD (Mondragón-Rodríguez et al., 2012; Kimura et al., 2014), likely dependent upon AMPA receptor internalization mediated by tau (Regan et al., 2015). These results suggest a physiologically important role for tau in synaptic transmission, as well as highlight a potential route of toxicity if the misprocessing of tau leads to signaling cascades inducing increased LTD in the hippocampus in AD. Furthermore, mutated tau was found to be associated with misprocessing of glutamate signaling and excitotoxicity *in vivo*, further supporting a role for tau in regulation of synaptic transmission (Hunsberger et al., 2015). Tau transgenic mice show alterations in spine and post-synaptic density volume, as well as basal plasticity changes measured by electrophysiology. The detection of defective myelination in axons from tauopathy mice suggests that signaling detriments may also be partially dependent on myelination errors (Maurin et al., 2014).

It is postulated that tau mediates the NMDA receptor through the tyrosine kinase, Fyn, a member of the Src family (Larson et al., 2012). The function of Fyn is to phosphorylate NR2B, a subunit of the NMDA receptor. Tau seems to have a crucial function at dendrites by targeting the kinase Fyn to postsynaptic compartments, resulting in the over-activation of NMDA receptors (Lee et al., 1998; Reynolds et al., 2008; Ittner et al., 2010). Tau or Aβ stabilization of NR2B with PSD95 enhances glutamatergic toxicity. This interaction seems to be mediated by phosphorylation of tau (Bhaskar et al., 2005; Reynolds et al., 2008; Usardi et al., 2011). However, dephosphorylated tau is able to cause cell death by activating muscarinic acetylcholine receptors with a higher affinity than acetylcholine (Gomez-Ramos et al., 2008, 2009; Diaz-Hernandez et al., 2010). In APP mice, reduction of Fyn prevented Aβ-mediated toxicity (Chin et al., 2004, 2005). The absence of tau or tau lacking the microtubule binding domain abolished Fyn targeting to dendritic spines, thus preventing memory deficits associated with Aβ. Moreover, inhibiting Fyn in a mouse model of AD led to a decrease in tau phosphorylation and reversal of memory deficits associated with a restoration of synapse density (Kaufman et al., 2015). Further, a double transgenic mouse generated by crossing mice overexpressing human APP (hAPP) and Htau mice exhibit accelerated cognitive impairment, enhanced aggregation of soluble and insoluble tau, as well as reduction of dendritic spines. Interestingly Fyn is upregulated only in the double transgenic but not in single transgenic Htau or hAPP, suggesting that the presence of Aβ and tau is required in order for Fyn to promote dendritic abnormalities(Chabrier et al., 2014). We have recently shown that reduction of tau oligomers by immunotherapy does not affect Fyn protein levels after treatment, but it does have effects on spine plasticity, suggesting that tau oligomers mediate a different pathway in dendritic spines (Castillo-Carranza et al., 2015). This may be true for tau oligomers specifically since it was previously reported that tau binding to Fyn is increased upon phosphorylation of tau (Mondragón-Rodríguez et al., 2012) particularly at AT8 or PHF1 sites, markers of NFTs, that seem to enhance Fyn SH3 binding to the proline-rich domain of tau (Bhaskar et al., 2005).

## Spread Of TAU Oligomers Across The Synapse

Functional tau has been detected in small concentrations at dendrites (Ittner et al., 2010). Under physiological conditions a small proportion of full-length tau is secreted to the extracellular space (Yamada et al., 2011) raising the question of whether extracellular tau has a physiological function. Tau has been found in cerebrospinal fluid (CSF) from healthy people (Ittner et al., 2010). In AD the levels of hyperphosphorylated tau in CSF correlated well with the progression of the disease. Moreover, tau was found in subcellular compartments responsible for protein trafficking and secretion such as autophagic vacuoles, endoplasmic reticulum, and Golgi apparatus (Tang et al., 2015). Microvesicle shedding and exosome release are some of the possible mechanisms that have been proposed to involve tau secretion from neurons. Exosomes refer to vesicles formed through budding of the endosomal membrane into larger vesicles termed multivesicular bodies (MVBs). Thus it is possible that once released from neurons, tau can eventually misfold, acquire a toxic function and become a potential source of seeds that can propagate throughout the brain. Extracellular tau released from ghost tangles or damaged neurons may become toxic to neighboring cells. However, recently Pooler et al. (2013) showed that propagation of tau pathology is an active process associated with synapses rather than release due to cell death. *In vivo*, microdialysis of mouse brains suggests that increasing neuronal and synaptic activity correlates with higher levels of extracellular tau (Yamada et al., 2011). Therefore, trans-synaptic communication is a possible avenue by which misfolded tau oligomers spread and compromise functional tau.

One of the most critical mechanisms under investigation in the field of neurodegeneration today is how tau pathology spreads from affected to unaffected brain regions. Many studies have suggested that oligomeric tau may be capable of propagating in the brain, inducing the misfolding of functional tau, reviewed by Gerson and Kayed (2013). While the mechanism is currently unknown and many different hypotheses exist, there has been some reported evidence for the spread of tau oligomers through the synapse. In combination with stereotypic staging of NFT pathology in AD that follows a transsynaptic pattern of spreading of pathology (Braak and Braak, 1991b), studies in animal models have provided direct evidence for this pathway.

Using a transgenic mouse that conditionally expresses tau in the area of the brain where Alzheimer’s disease pathology first arises—the entorhinal cortex—researchers have shown that tau appears to spread between synaptically connected brain regions, suggesting a potential role of the synapse in pathological tau transport (de Calignon et al., 2012; Liu et al., 2012). After thorough analysis of mice injected with tau aggregates in the hippocampus, it was found that tau spreading occurred in none of 20 neighboring brain regions analyzed and was only found in synaptically connected areas (Ahmed et al., 2014). In order to determine whether tau oligomeric aggregates are capable of transporting between the axonal and somatodendritic compartments of the neuron to allow for synaptic transport of tau taken up in the cell in disease, neurons were cultured in microfluidic chambers and treated with tau oligomers. Researchers found that oligomeric tau was able to spread both anterogradely and retrogradely between cellular compartments, though tau monomers and fibrils were not able to enter the cell when administered in the media (Wu et al., 2013). Further evidence of the trans-synaptic mechanism for the spread of tau aggregates is the presence of phosphorylated tau at the synapse in AD brain that correlates with dysfunction of the ubiquitin proteasome system. This accumulation of tau unable to be degraded may account for the spread of tau through the synapse (Tai et al., 2012). Moreover, stimulation of neuronal firing through the activation of AMPA receptors led to an increase in extracellular tau in cultured cells, while levels were decreased when pharmacological agents reducing synaptic vesicle release and neuronal activity were applied (Pooler et al., 2013). In order to confirm the importance of synaptic contacts in the transfer of tau aggregates between cells, neurons expressing synaptogenic adhesion proteins were cultured to induce the formation of synapses. When compared to control cells, neurons with heightened synaptic formation also increased the uptake and spreading of tau aggregates after treatment. Moreover, inhibiting the formation of synapses, as well as neuronal activity led to a decrease in the ability of tau aggregates administered to cells to transport between neurons (Calafate et al., 2015).

## Conclusion

In spite of all the evidence suggesting a toxic role of tau, therapeutic interventions have been focused on targeting Aβ in preclinical and clinical studies. Although preclinical studies showed very exciting results, clinical trials did not prevent the progression of cognitive decline. It seems likely that Aβ initiates a cascade of events that at a certain stage becomes irreversible, thereby making Aβ removal insufficient to avert cognitive decline. A secondary pathological event that causes dementia and has the potential to become independent of Aβ pathology is the formation of toxic tau oligomers. These structures are able to self-propagate, spread through synapses and induce synaptic dysfunction. Thus, understanding the mechanisms by which tau oligomers spread throughout the synapse may be critical for the design of novel therapeutic strategies to treat AD.

## Conflict of Interest Statement

The authors declare that the research was conducted in the absence of any commercial or financial relationships that could be construed as a potential conflict of interest.
